# Association between Intake of Edible Mushrooms and Algae and the Risk of Cognitive Impairment in Chinese Older Adults

**DOI:** 10.3390/nu16050637

**Published:** 2024-02-25

**Authors:** Yun Yang, Danni Zhu, Ran Qi, Yanchun Chen, Baihe Sheng, Xinyu Zhang

**Affiliations:** 1 School of Public Health, Tianjin Medical University, Tianjin 300070, China; yunyang@tmu.edu.cn (Y.Y.); zhudanni781@tmu.edu.cn (D.Z.); qiran@tmu.edu.cn (R.Q.); chenyanchun@tmu.edu.cn (Y.C.); shengbaihe@tmu.edu.cn (B.S.); 2 Tianjin Key Laboratory of Environment, Nutrition and Public Health, Tianjin Medical University, Tianjin 300070, China

**Keywords:** edible mushrooms, algae, cognitive impairment, older adults

## Abstract

Previous studies have investigated the association between diet and cognitive impairment, yet there is limited investigation into the link between edible mushrooms and algae intake and cognitive decline. This study aims to explore the association between edible mushrooms and algae intake and the risk of cognitive impairment in individuals aged 65 years and above in China. Cross-sectional data from the 2018 Chinese Longitudinal Healthy Longevity Survey (CLHLS) formed the basis of this study. Edible mushrooms and algae intake was evaluated using a simplified food frequency questionnaire (FFQ) and cognitive function was assessed using the Mini-Mental State Examination (MMSE). A binary logistic regression model was used to evaluate odds ratios (ORs) and 95% confidence intervals (CIs), with subgroup analysis conducted. Among 14,150 older adults, the average age was (85.33 ± 11.55), with a cognitive impairment prevalence of 22.7; multi-model adjustments showed a 25.3% lower probability of cognitive impairment for those occasionally consuming edible mushrooms and algae (OR: 0.747, 95% CI: 0.675~0.826). Furthermore, a 29% lower risk was observed in those with daily intake (OR: 0.710, 95% CI: 0.511~0.987). Subgroup analysis demonstrated significant risk reduction in women (OR: 0.589, 95% CI: 0.375~0.925, *p* = 0.022), individuals with disability in activities of daily living (OR: 0.568, 95% CI: 0.367~0.878, *p* = 0.011), and those with low social activity levels (OR: 0.671, 95% CI: 0.473~0.950, *p* = 0.025). This study concludes that edible mushrooms and algae intake significantly impacts the risk of cognitive impairment in older adults. These results provide insights and impetus for further research into this area. Additional cohort studies or intervention trials are necessary to confirm the potential benefits of edible mushrooms and algae in promoting cognitive health.

## 1. Introduction

Aging has become an important global public health challenge, particularly in China. In the context of escalating population aging, cognitive decline has emerged as a pervasive feature of the aging trajectory. Throughout the process of aging, there is a progressive deterioration in various aspects of cognitive function, encompassing memory, language proficiency, attention span, and executive functioning. This decline in cognitive abilities not only impacts the individual’s quality of life and functional independence in older adults but also gives rise to significant societal and economic implications. In 2020, the prevalences of cognitive decline and dementia among people aged 60 and above in China were 15.54% and 6.04%, respectively [1,2,3]. With advancing age, individuals are prone to neurodegeneration, leading to mild cognitive impairment that may progress to severe cognitive impairment, dementia, or Alzheimer’s disease (AD). This progression often results in a loss of the ability to complete daily tasks, leading to an inability to live independently [4,5,6]. Cognitive impairment serves as a prodromal stage of dementia, presenting an opportunity to implement measures to prevent dementia [7]. To reduce the risk of cognitive impairment, new and effective preventive methods are needed, including the application of dietary strategies.

Numerous studies have investigated the relationship between single diets or dietary patterns and cognitive impairment [8,9,10,11]. These studies revealed that adherence to a “vegetable-pork” diet, a Mediterranean-style diet, tea consumption, and increased intake of vegetables and mushrooms were all significantly associated with better cognitive function, whereas animal-based dietary patterns were identified as a potential risk factor for cognitive decline [12]. However, despite the growing research interest in exploring the link between dietary intake and cognitive health, there remains a paucity of evidence regarding the association between edible mushrooms and algae consumption and cognitive impairment. Edible mushrooms and algae, such as mushrooms, fungus, snow fungus, kelp, seaweed, etc., are rich sources of nutrients and potent antioxidants, including protein, βglucan, minerals, vitamins, and ergothioneine [13,14,15], which are believed to exert a beneficial effect on preserving brain health.

Existing research has indicated a correlation between mushroom consumption and a reduced risk of cognitive impairment. However, the literature on the association between algal food intake and cognitive impairment remains limited. Moreover, studies investigating the relationship between edible mushrooms, algae food, and cognitive impairment have predominantly focused on populations in the United States, Japan, Singapore, and other regions [16,17,18], with relatively little research on the Chinese population and the older persons population, rather than relatively little research on the Chinese older persons population. Furthermore, research involving edible mushrooms and algae is notably scarce. Therefore, there is a pressing need for additional studies in the Chinese population to deepen our understanding of the potential impact of edible mushrooms and algae on the risk of cognitive impairment.

This study aims to address this knowledge gap, utilizing the representative Chinese Longitudinal Healthy Longevity Survey (CLHLS) database to analyze the association between edible mushrooms and algae intake and cognitive impairment in Chinese older adults aged 65 years and above. The goal is to draw attention to a healthy diet and provide the scientific basis for the prevention and control of cognitive impairment.

## 2. Objects and Methods

### 2.1. Study Population

This study utilized cross-sectional data from the CLHLS in 2018. The CLHLS is an ongoing prospective cohort study of Chinese adults aged 65 years and older, established in 1998 using multi-stage cluster sampling across 23 provinces in China, representing approximately 85% of the Chinese population. The CLHLS received approval from the Biomedical Ethics Committee of Peking University, China (IRB00001052–13074, Date: 9 November 2021), and all participants signed informed consent.

**Figure 1:**
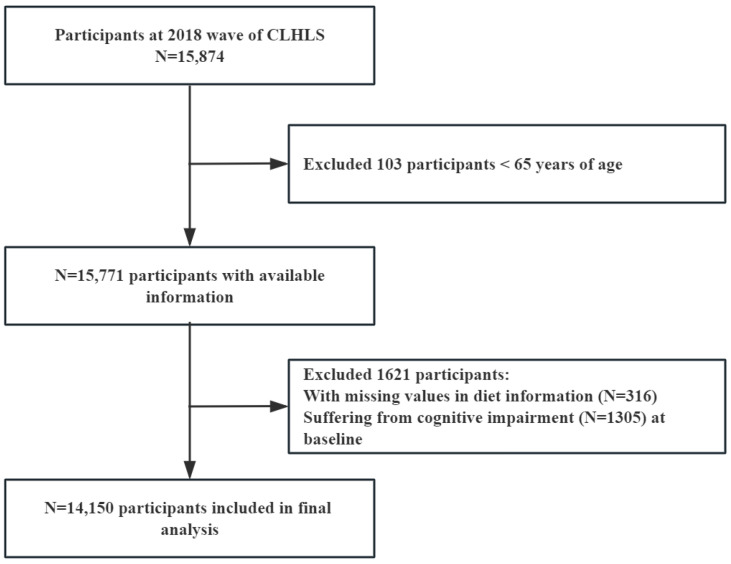
Flowchart of participant screening.

In 2018, a total of 15,874 participants completed the CLHLS survey. For this study, 103 people under 65 were excluded, and 1621 participants who lacked information about edible mushrooms and algae intake and cognitive function were excluded. Finally, 14,150 people were included in this study (Figure 1).

### 2.2. Intake of Edible Mushrooms and Algae

The CLHLS database collected self-reported dietary intake data from participants via a simplified food frequency questionnaire (FFQ), which includes 22 food group items. Previous studies have established the reliability and validity of this simplified FFQ for Chinese subjects, and it has been extensively employed in various research investigations [19,20]. In the simplified food frequency questionnaire, edible mushrooms and algae mainly included mushrooms, fungus, snow fungus, kelp, seaweed, etc. The survey inquired about the frequency of edible mushrooms and algae consumption through direct one-on-one interviews, such as “Do you regularly consume edible mushrooms and algae?”. Participants were asked to indicate their consumption frequency using a five-level categorization: “Almost every day”, “At least once a week, but not daily”, “At least once a month, but not weekly”, “Occasionally, but not monthly”, and “Rarely or never”. In the process of analysis, the consumption frequency of edible mushrooms and algae in the questionnaire was divided into three categories: daily (almost daily), occasionally (at least once a week/at least once a month/sometimes), and never (rarely/never).

### 2.3. Assessment of Cognitive Impairment

The Chinese version of the Mini-Mental State Examination (MMSE) evaluated five dimensions: general ability, reaction ability, attention and calculation ability, recall ability, and language ability. Apart from the inquiry “Can you tell me what people can eat?”, other questions were scored, granting 1 point for correct answers and 0 points for incorrect or unanswerable ones, contributing to a cumulative score ranging from 0 to 30 points. Following established research [21], since the MMSE score may be influenced by the level of education, cognitive impairment was defined in the case of illiteracy as an MMSE score of ≤17; in the case of primary education as an MMSE score of ≤20 points; and for secondary education and above, an MMSE score of ≤24 points.

### 2.4. Assessment of Covariates

Demographic characteristics, lifestyle and behavior, and population health status served as covariates in this study. The adjusted demographic characteristics comprised age (years), sex (male or female), type of residence (urban or rural), marital status (with or without spouse), and education (illiteracy, primary, or secondary and above). Lifestyle and behaviors encompassed sleep duration (≤6 h, 7–8 h, or ≥9 h), smoking (current, former, or never), alcohol consumption (current, former, or never), vegetable intake (yes or no), fruit intake (yes or no), and social activity level (low or high). Health status covered body mass index (BMI; underweight, normal, overweight or obese), disability in activities of daily living (yes or no), chronic diseases (hypertension, diabetes, heart disease, stroke, and cerebrovascular disease; yes or no).

The assessment of social activity level involved eight activities in the questionnaire: Tai Chi, square dancing, growing flowers and raising pets, reading books and newspapers, raising poultry and livestock, playing cards or mahjong, watching TV and listening to radio, and participating in social activities. Each activity received a score on a scale of 1 (never), 2 (sometimes), and 3 (almost daily), resulting in a cumulative score ranging from 8 to 24. A score below 14 was defined as a low level of social activity [11]. The evaluation of activities of daily living (ADLs) was assessed using the Katz Index and the ADL Scale as a reference. Key assessment indicators included bathing, dressing, toilet use, indoor activities, control of defecation, and eating, evaluated based on the need for assistance. When one or more indicators required support, they were classified as a disability in activities of daily living [22].

### 2.5. Statistical Analysis

Baseline characteristics were summarized as percentages of categorical variables based on the frequency of edible mushrooms and algae intake. The relationship between characteristics and edible mushrooms and algae intake was analyzed using the Wilcoxon signed rank test and the Kruskal–Wallis test. With cognitive impairment as the dependent variable and the frequency of edible mushrooms and algae intake as the independent variable, the association between edible mushrooms and algae intake and cognitive impairment was analyzed by binary logistic regression model. The model adjusted for several covariables to control the influence of confounding factors. Model 1 controlled for the age and sex of the participants. Model 2 was further adjusted for residence, marital status, and education. Model 3 additionally controlled for sleep duration, smoking, alcohol consumption, vegetable intake, fruit intake, level of social activity, BMI, disability in activities of daily living, and chronic diseases (hypertension, diabetes, heart disease, stroke, and cerebrovascular disease). Multiple imputations by chained equations were conducted to fill in missing values of the covariates. The fully adjusted model was also employed in subgroup analyses to investigate whether the association between edible mushrooms and algae intake and cognitive impairment differed by sex, disability in activities of daily living, and level of social activity. All analyses were performed using SPSS 26.0 and R 4.3.1 statistical software. Test level α = 0.05.

## 3. Results

A total of 14,150 subjects with an average age of (85.33 ± 11.55) years were included in this study, including 6213 males (43.9%), 7937 females (56.1%), 7911 (55.9%) urban dwellers, and 7207 (50.9%) subjects who were illiterate. Cognitive impairment occurred in 3208 patients (22.7%). The proportions of occasional and daily consumption of edible mushrooms and algae were 54.8% (n = 7751) and 2.9% (n = 409), respectively. Statistically significant differences in the frequency of edible mushrooms and algae intake were observed by age, sex, residence, education, marital status, sleep duration, smoking, alcohol consumption, fruit and vegetable intake, social activity level, BMI, disability in activities of daily living, hypertension, diabetes, heart disease, and stroke and cerebrovascular disease (all *p* < 0.05) (Table 1).

The binary logistic regression analysis, with cognitive impairment as the dependent variable and the frequency of edible mushrooms and algae intake as the independent variable, demonstrated that after adjusting for age and sex (Model 1), the probability of cognitive impairment was reduced by 32.7% (OR: 0.673, 95% CI: 0.618~0.733) and 38.7% (OR: 0.613, 95% CI: 0.459~0.820), respectively (both *p* < 0.05), in the older persons who occasionally and daily ate edible mushrooms and algae compared with those who never ate them. Further adjustments were performed for residence, marital status, and education in Model 2 which showed that the probability of cognitive impairment in older adults who consumed edible mushrooms and algae occasionally and daily was reduced by 30.2% (OR: 0.698, 95% CI: 0.639~0.763) and 32.6% (OR: 0.674, 95% CI: 0.501~0.909), respectively (both *p* < 0.05). Subsequent adjustments in Model 3, incorporating variables such as sleep duration, smoking, alcohol consumption, vegetable intake, fruit intake, social activity level, BMI, disability in activities of daily living, hypertension, diabetes, heart disease, and stroke and cerebrovascular disease, revealed 25.3% (OR: 0.747, 95% CI: 0.675~0.826) and 29% (OR: 0.710, 95% CI: 0.511~0.987) reductions in the probability of cognitive impairment in those who consumed edible mushrooms and algae occasionally and daily, respectively (both *p* < 0.05). Daily intake of edible mushrooms and algae was associated with a lower risk of cognitive impairment than occasional consumption (Table 2).

In the subgroup analysis, distinctions based on sex, disability in activities of daily living, and social activity level were examined. Following adjustment for relevant confounding factors, the occasional consumption of edible mushrooms and algae (OR: 0.747, 95% CI: 0.632~0.883, *p* = 0.001) reduced the risk of cognitive impairment in men. Conversely, in women, both occasional and daily consumption of edible mushrooms and algae reduced the likelihood of cognitive impairment. Notably, the probability value was higher among those who consumed edible mushrooms and algae daily, standing at 41.1% (OR: 0.589, 95% CI: 0.375–0.925, *p* = 0.022).

Among individuals without disabilities in activities of daily living, occasional consumption of edible mushrooms and algae correlated with a 23% lower risk of cognitive impairment (OR: 0.770, 95% CI: 0.669~0.886, *p* < 0.001). Conversely, in individuals with disability in activities of daily living, whether the consumption was occasional or daily, it led to a reduction in the probability of cognitive impairment. For those with daily consumption of edible mushrooms and algae, this probability value was higher, reaching 43.2% (OR: 0.568, 95% CI: 0.367–0.878, *p* = 0.011).

**Figure 2:**
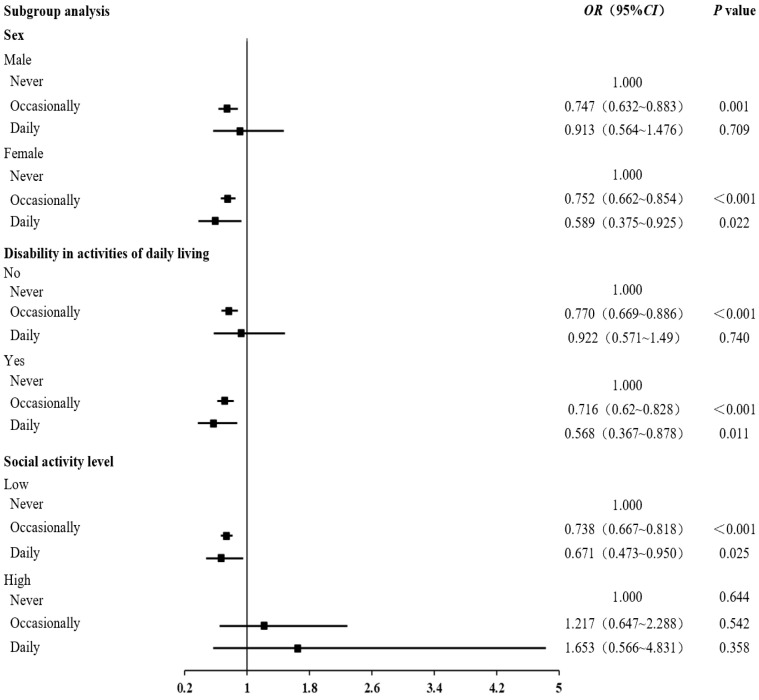
Subgroup analysis. The sex subgroup was analyzed by removing the sex variable from Model 3, the disability in activities of daily living subgroup was analyzed by removing the disability in activities of daily living variable from Model 3, and the social activity level subgroup was analyzed by removing the social activity level variable from Model 3.

Among individuals with low levels of social activity, occasional consumption of edible mushrooms and algae demonstrated an effective risk reduction of 26.2% (OR: 0.738, 95% CI: 0.667~0.818, *p* < 0.001). Meanwhile, daily consumption of edible mushrooms and algae was associated with a more substantial risk reduction of 32.9% (OR: 0.671, 95% CI: 0.473~0.950, *p* = 0.025). However, for individuals with higher levels of social activity, there was no statistically significant association between the consumption of edible mushrooms and algae and cognitive impairment (Figure 2).

## 4. Discussion

This study’s findings indicate that both occasional and daily consumption of edible mushrooms and algae are associated with a lower risk of cognitive impairment compared to those who never consume these foods. Furthermore, daily consumption of edible mushrooms and algae was associated with a decreased risk of cognitive impairment in comparison to occasional edible mushrooms and algae consumption. Subgroup analysis revealed that in women, individuals with disability in activities of daily living, and those with low levels of social activity, the daily consumption of edible mushrooms and algae significantly reduced the risk of cognitive impairment.

Our results basically align with prior studies. Shu Zhang et al. [17] demonstrated a significant association between regular consumption of mushrooms and a reduced risk of dementia among individuals aged 65 years and above in a Japanese cohort study. Specifically, the consumption of mushrooms 1–2 times per week and ≥3 times per week was linked to a reduced risk of dementia. In a recent investigation utilizing data from the Diet and Chronic Disease Study cohort within a European population, researchers discovered that individuals who consumed mushrooms exhibited superior performance in various cognitive domains compared to non-consumers. Moreover, a dose–response relationship was identified, indicating that those who consumed one or more servings of mushrooms per week demonstrated the highest cognitive scores [15]. In the research conducted by Kim et al. [23], the Morris water maze experiment and choline acetyltransferase immunohistochemistry were employed to assess the cognitive enhancement effects of Tremella tremella on scopolamine-induced amnesia in rats. The results demonstrated that oral tremella therapy for 14 consecutive days could reverse learning and memory deficits induced by scopolamine. Another study suggested that tremella water extract promoted neurogenesis in neuron-cultured cells, improving spatial learning and memory tasks in rats [24]. Additionally, a study on the cognitive performance of plant-based food intake in older adults found that those who consumed mushrooms performed better on six cognitive tests than those who did not, and the dose–response relationship between mushrooms and cognitive ability tended to be linear [25]. In their study on algae and cognitive impairment, Reid et al. [26] conducted a randomized, double-blind, controlled study to investigate the potential benefits of algae consumption on cognitive dysfunction in elderly individuals. The study enrolled 60 moderately active participants, and the results revealed that kelp intake played a significant role in preventing aging and in the degradation of short-term memory and physical function.

Previous studies have consistently highlighted that being female, disability in activities of daily living, and low levels of social activity are risk factors for cognitive impairment. Qilin Zhang et al. [27] demonstrated that the mean cognitive scores of male older adults in China from 2005 to 2014 (27.87, 27.07, 26.68, 25.52) were consistently higher than those of female older adults (26.32, 25.33, 24.47, 22.69), with the cognitive decline of males (2.35) significantly lower than in females (3.63). In their investigation of the pathological mechanisms involved, Yan Yan et al. [28] revealed that the expression levels of the protein USP11 in the female brain exceeded those observed in males. This disparity contributed to a higher prevalence of abnormal tau protein deposition in women compared to men, consequently elevating the susceptibility of women to Alzheimer’s disease. Zhou Chuyi et al. [29] found a significant negative correlation (−0.355) between the ability to perform daily living scores of older adults and the MMSE scores. A prospective cohort study in China revealed a 19% increase in the incidence of cognitive impairment in those with lower levels of social activity relative to those with high levels of social activity [27]. In the subgroup analysis results of this study, the daily consumption of edible mushrooms and algae significantly reduced the risk of cognitive impairment in women, people with disability in activities of daily living, and people with low levels of social activity. Therefore, this diet can be considered as an effective intervention.

The consumption of edible mushrooms and algae has potential benefits for cognitive function. Edible mushrooms contain various natural free radical scavengers, including polysaccharides [30], polyphenols [31], vitamins [32,33], and ergosterol [34], providing beneficial effects through these scavengers [35,36,37]. Bioactive compounds in edible mushrooms can protect the brain against neurodegeneration by inhibiting the production of amyloid, phosphorylated tau, and acetylcholinesterase [38]. In addition, the potent antioxidant ergothioneine, a sulfur-containing amino acid that can only be obtained from dietary sources is exceptionally high in edible mushrooms, with mushrooms having the highest concentration among dietary ingredients [39,40,41,42,43]. Some studies suggest that edible mushrooms may have a protective effect against diseases associated with the risk of cognitive impairment, such as atherosclerosis [44,45], hypertension [46,47], and diabetes [48,49], thereby reducing the risk of cognitive impairment. While the exact mechanism by which algal food is associated with cognitive impairment remains unclear, there is evidence suggesting that algal food might counteract age-related degradation by increasing antioxidant capacity [50]. In addition, algae food is rich in vitamin B12, which has the potential to activate brain nerves and prevent memory decline [51,52].

The strength of this study lies in its use of nationally representative data from the Chinese older persons population, providing a strong basis for verifying the association between edible mushrooms and algae intake and cognitive impairment. Edible mushrooms and algae are unique to the Chinese diet and are essential components of the daily food intake of Chinese people. This study conducted comprehensive covariate adjustments and subgroup analyses to minimize potential confounders that could interfere with the results. Subgroup analysis, focusing on social attributes and daily living ability, adds practical guiding significance to the findings. However, this study has certain limitations. First, as a cross-sectional study, it cannot establish a causal relationship. Secondly, the reliance on self-reported information introduces a potential for bias. Thirdly, the simplified food frequency questionnaire used by the CLHLS only collected the frequency of food consumption and failed to calculate the association of intake with cognitive impairment. Lastly, despite extensive adjustments for confounders, confounding bias may still be present.

## 5. Conclusions

Using nationally representative data from older Chinese adults, our investigation indicates that both occasional and daily consumption of edible mushrooms and algae are linked to a diminished risk of cognitive impairment compared to those who abstain from such consumption. Furthermore, the risk of cognitive impairment is observed to be lower in cases of daily ingestion of edible mushrooms and algae in comparison to occasional consumption. Subgroup analysis reveals a noteworthy reduction in the risk of cognitive impairment among specific demographics, including women, individuals with disabilities in activities of daily living, and those with limited social activity, who incorporate a daily regimen of edible mushrooms and algae into their diet. The outcomes of this study underscored the significant impact of edible mushrooms and algae intake on the risk of cognitive impairment in older persons. These results provide insights and impetus for further research into this area. Such investigations, ideally conducted through cohort studies or intervention trials, will contribute to a more comprehensive understanding of the observed associations and their implications for cognitive health in the aging population.

## Figures and Tables

**Table 1:** Baseline characteristics according to edible mushrooms and algae intake frequency.

Characteristics	Total	Edible Mushrooms and Algae Intake Frequency			*p* Value
		Never	Occasionally	Daily	
N	14,150	5990 (42.3)	7751 (54.8)	409 (2.9)	
Age (years)					<0.001
<80	4898 (34.6)	1760 (29.4)	2954 (38.1)	184 (45.0)	
≥80	9252 (65.4)	4230 (70.6)	4797 (61.9)	225 (55.0)	
Sex					<0.001
Male	6213 (43.9)	2503 (41.8)	3515 (45.3)	195 (47.7)	
Female	7937 (56.1)	3487 (58.2)	4236 (54.7)	214 (52.3)	
Residence					<0.001
Urban	7911 (55.9)	2773 (46.3)	4822 (62.2)	316 (77.3)	
Rural	6239 (44.1)	3217 (53.7)	2929 (37.8)	93 (22.7)	
Education					<0.001
Illiterate	7207 (50.9)	3714 (62.0)	3385 (43.7)	108 (26.4)	
Primary	4418 (31.2)	1721 (28.7)	2583 (33.3)	114 (27.9)	
Secondary and above	2525 (17.8)	555 (9.3)	1783 (23.0)	187 (45.7)	
Marital status					<0.001
Without spouse	8291 (58.6)	3814 (63.7)	4294 (55.4)	183 (44.7)	
With spouse	5859 (41.4)	2176 (36.3)	3457 (44.6)	226 (55.3)	
Sleep duration					<0.001
≤6 h	5303 (37.5)	2419 (40.4)	2737 (35.3)	147 (35.9)	
7–8 h	5053 (35.7)	1909 (31.9)	2984 (38.5)	160 (39.1)	
≥9 h	3794 (26.8)	1662 (27.7)	2030 (26.2)	102 (24.9)	
Smoking					<0.001
Never	9984 (70.6)	4331 (72.3)	5369 (69.3)	284 (69.4)	
Former	2072 (14.6)	774 (12.9)	1229 (15.9)	69 (16.9)	
Current	2094 (14.8)	885 (14.8)	1153 (14.9)	56 (13.7)	
Alcohol consumption					<0.001
Never	10,546 (74.5)	4591 (76.6)	5660 (73.0)	295 (72.1)	
Former	1612 (11.4)	659 (11.0)	901 (11.6)	52 (12.7)	
Current	1992 (14.1)	740 (12.4)	1190 (15.4)	62 (15.2)	
Fruit intake					<0.001
No	3511 (24.8)	2043 (34.1)	1421 (18.3)	47 (11.5)	
Yes	10,639 (75.2)	3947 (65.9)	6330 (81.7)	362 (88.5)	
Vegetable intake					<0.001
No	545 (3.9)	382 (6.4)	157 (2.0)	6 (1.5)	
Yes	13,605 (96.1)	5608 (93.6)	7594 (98.0)	403 (98.5)	
Social activity level					<0.001
Low	12,171 (86.0)	5527 (92.3)	6372 (82.2)	272 (66.5)	
High	1979 (14.0)	463 (7.7)	1379 (17.8)	137 (33.5)	
BMI (kg/m^2^)					<0.001
Underweight	2452 (17.3)	1268 (21.2)	1135 (14.6)	49 (12.0)	
Normal	7344 (51.9)	3220 (53.8)	3912 (50.5)	212 (51.8)	
Overweight or obese	4354 (30.8)	1502 (25.1)	2704 (34.9)	148 (36.2)	
Disability in activities of daily living					0.009
No	10,154 (71.8)	4229 (70.6)	5627 (72.6)	298 (72.9)	
Yes	3996 (28.2)	1761 (29.4)	2124 (27.4)	111 (27.1)	
Hypertension					<0.001
No	8228 (58.1)	3785 (63.2)	4259 (54.9)	184 (45.0)	
Yes	5922 (41.9)	2205 (36.8)	3492 (45.1)	225 (55.0)	
Diabetes					<0.001
No	12,784 (90.3)	5614 (93.7)	6829 (88.1)	341 (83.4)	
Yes	1366 (9.7)	376 (6.3)	922 (11.9)	68 (16.6)	
Heart disease					
No	11,709 (82.7)	5179 (86.5)	6223 (80.3)	307 (75.1)	<0.001
Yes	2441 (17.3)	811 (13.5)	1528 (19.7)	102 (24.9)	
Stroke and cerebrovascular disease					<0.001
No	12,591 (89.0)	5438 (90.8)	6814 (87.9)	339 (82.9)	
Yes	1559 (11.0)	552 (9.2)	937 (12.1)	70 (17.1)	

**Table 2:** Association between edible mushrooms and algae intake and cognitive impairment.

Model	Never	Occasionally		Daily	
		OR (95% CI)	*p* Value	OR (95% CI)	*p* Value
Model 1 ^a^	1.00 (Ref.)	0.67 (0.62, 0.73)	<0.001	0.61 (0.46, 0.82)	0.001
Model 2 ^b^	1.00 (Ref.)	0.70 (0.64, 0.76)	<0.001	0.67 (0.50, 0.91)	0.010
Model 3 ^c^	1.00 (Ref.)	0.75(0.68, 0.83)	<0.001	0.71 (0.51, 0.99)	0.041

^a^ Adjusted for age and sex; ^b^ Model 1+ adjusted for residence, marital status, and education; ^c^ Model 2+ adjusted for sleep duration, smoking, alcohol consumption, vegetable intake, fruit intake, social activity level, BMI, disability in activities of daily living, and chronic diseases (hypertension, diabetes, heart disease, and stroke and cerebrovascular disease).

## Data Availability

The data I used in my research came from the CLHLS database, which is a public database that anyone can find and use on the website. This is the database url: https://opendata.pku.edu.cn/.

## References

[1] Jia L., Du Y., Chu L., Zhang Z., Li F., Lyu D., Li Y., Zhu M., Jiao H., Song Y. (2020). Prevalence, risk factors, and management of dementia and mild cognitive impairment in adults aged 60 years or older in China: A cross-sectional study. Lancet Public Health.

[2] Pang S.J., Jia S.S., Man Q.Q., Song S., Li Y.Q., Song P.K., Zhao W.H., Zhang J. (2017). Dietary Cholesterol in the Elderly Chinese Population: An Analysis of CNHS 2010–2012. Nutrients.

[3] Prince M. (2015). World Alzheimer Report 2015: The Global Impact of Dementia. https://www.alzint.org/resource/world-alzheimer-report-2015/.

[4] Bredesen D.E., Amos E.C., Canick J., Ackerley M., Raji C., Fiala M., Ahdidan J. (2016). Reversal of cognitive decline in Alzheimer’s disease. Aging.

[5] Giebel C.M., Sutcliffe C., Challis D. (2015). Activities of daily living and quality of life across different stages of dementia: A UK study. Aging Ment. Health.

[6] Whitehouse P.J. (1999). Alzheimer’s disease: Past, present, and future. Eur. Arch. Psychiatry Clin. Neurosci..

[7] Rabin L.A., Smart C.M., Amariglio R.E. (2017). Subjective Cognitive Decline in Preclinical Alzheimer’s Disease. Annu. Rev. Clin. Psychol..

[8] Zhang X., Huang F., Zhang J., Wei Y., Bai J., Wang H., Jia X. (2023). Association between Micronutrient-Related Dietary Pattern and Cognitive Function among Persons 55 Years and Older in China: A Longitudinal Study. Nutrients.

[9] Gou R., Qin J., Pang W., Cai J., Luo T., He K., Xiao S., Tang X., Zhang Z., Li Y. (2023). Correlation between dietary patterns and cognitive function in older Chinese adults: A representative cross-sectional study. Front. Nutr..

[10] Solfrizzi V., Custodero C., Lozupone M., Imbimbo B.P., Valiani V., Agosti P., Schilardi A., D’Introno A., La Montagna M., Calvani M. (2017). Relationships of Dietary Patterns, Foods, and Micro- and Macronutrients with Alzheimer’s Disease and Late-Life Cognitive Disorders: A Systematic Review. J. Alzheimers Dis..

[11] Yao Y., Chen H., Chen L., Ju S.-Y., Yang H., Zeng Y., Gu D., Ng T.P. (2021). Type of tea consumption and depressive symptoms in Chinese older adults. BMC Geriatr..

[12] Hu W., Zhang H., Ni R., Cao Y., Fang W., Chen Y., Pan G. (2023). Interaction between the animal-based dietary pattern and green space on cognitive function among Chinese older adults: A prospective cohort study. Int. J. Hyg. Environ. Health.

[13] Jo Feeney M., Miller A.M., Roupas P. (2014). Mushrooms-Biologically Distinct and Nutritionally Unique: Exploring a “Third Food Kingdom”. Nutr. Today.

[14] Fulgoni V.L., Agarwal S. (2021). Nutritional impact of adding a serving of mushrooms on usual intakes and nutrient adequacy using National Health and Nutrition Examination Survey 2011–2016 data. Food Sci. Nutr..

[15] Cha S., Bell L., Williams C.M. (2024). The Relationship between Mushroom Intake and Cognitive Performance: An Epidemiological Study in the European Investigation of Cancer-Norfolk Cohort (EPIC-Norfolk). Nutrients.

[16] Ba D.M., Gao X., Al-Shaar L., Muscat J.E., Chinchilli V.M., Ssentongo P., Beelman R.B., Richie J. (2022). Mushroom intake and cognitive performance among US older adults: The National Health and Nutrition Examination Survey, 2011–2014. Br. J. Nutr..

[17] Zhang S., Tomata Y., Sugiyama K., Sugawara Y., Tsuji I. (2017). Mushroom Consumption and Incident Dementia in Elderly Japanese: The Ohsaki Cohort 2006 Study. J. Am. Geriatr. Soc..

[18] Feng L., Cheah I.K.-M., Ng M.M.-X., Li J., Chan S.M., Lim S.L., Mahendran R., Kua E.-H., Halliwell B. (2019). The Association between Mushroom Consumption and Mild Cognitive Impairment: A Community-Based Cross-Sectional Study in Singapore. J. Alzheimers Dis..

[19] Zhao W.-H., Huang Z.-P., Zhang X., He L., Willett W., Wang J.-L., Hasegawa K., Chen J.-S. (2010). Reproducibility and validity of a Chinese Food Frequency Questionnaire. Biomed. Environ. Sci..

[20] Zhao W., Hasegawa K., Chen J. (2002). The use of food-frequency questionnaires for various purposes in China. Public Health Nutr..

[21] Nie H., Li H., Yang L., Hu B., Sun L., Sheng J., Zhang D., Chen G., Cheng B., Meng X. (2023). Association of Nighttime Sleep Duration with Cognitive Impairment among Community-dwelling Older Adults. Chin. Gen. Pract..

[22] Li M., Zhang C., Zhao H., Zheng X., Lu J., Chang Y., Cai Y. (2019). Disability Status and Its Influencing Factors among Empty Nesters and Non-empty Nesters in China. Chin. Gen. Pract..

[23] Kim J.H., Ha H.-C., Lee M.-S., Kang J.-I., Kim H.-S., Lee S.-Y., Pyun K.-H., Shim I. (2007). Effect of Tremella fuciformis on the neurite outgrowth of PC12h cells and the improvement of memory in rats. Biol. Pharm. Bull..

[24] Park H.J., Shim H.S., Ahn Y.H., Kim K.S., Park K.J., Choi W.K., Ha H.C., Kang J.I., Kim T.S., Yeo I.H. (2012). Tremella fuciformis enhances the neurite outgrowth of PC12 cells and restores trime-thyltin-induced impairment of memory in rats via activation of CREB transcription and cholinergic systems. Behav. Brain Res..

[25] Nurk E., Refsum H., Drevon C.A., Tell G.S., Nygaard H.A., Engedal K., Smith A.D. (2010). Cognitive performance among the elderly in relation to the intake of plant foods. The Hordaland Health Study. Br. J. Nutr..

[26] Reid S.N.S., Ryu J.-K., Kim Y., Jeon B.H. (2018). The Effects of Fermented *Laminaria japonica* on Short-Term Working Memory and Physical Fitness in the Elderly. Evid.-Based Complement. Altern. Med..

[27] Zhang Q., Wu Y., Han T., Liu E. (2019). Changes in Cognitive Function and Risk Factors for Cognitive Impairment of the Elderly in China: 2005–2014. Int. J. Environ. Res. Public Health.

[28] Yan Y., Wang X., Chaput D., Shin M.-K., Koh Y., Gan L., Pieper A.A., Woo J.-A., Kang D.E. (2022). X-linked ubiquitin-specific peptidase 11 increases tauopathy vulnerability in women. Cell.

[29] Zhou C.Y., Liu W.W., Guan Z.Y., Tang S.Y. (2020). Correlation analysis of activities of daily living and cognitive function of the elderly in senior care centers. Chin. J. Prev. Med..

[30] Jayakumar T., Thomas P.A., Mathivanan I., Geraldine P. (2010). An extract of the oyster mushroom, *Pleurotus ostreatus*, increases catalase gene expression and reduces protein oxidation during aging in rats. Zhong Xi Yi Jie He Xue Bao.

[31] Lam Y.S., Okello E.J. (2015). Determination of Lovastatin, β-glucan, Total Polyphenols, and Antioxidant Activity in Raw and Processed Oyster Culinary-Medicinal Mushroom, Pleurotus ostreatus (Higher Basidiomycetes). Int. J. Med. Mushrooms.

[32] Bennett L., Kersaitis C., Macaulay S.L., Münch G., Niedermayer G., Nigro J., Payne M., Sheean P., Vallotton P., Zabaras D. (2013). Vitamin D2-enriched button mushroom (*Agaricus bisporus*) improves memory in both wild type and APPswe/PS1dE9 transgenic mice. PLoS ONE.

[33] Vamanu E. (2014). Antioxidant properties of mushroom mycelia obtained by batch cultivation and tocopherol content affected by extraction procedures. BioMed Res. Int..

[34] Kobori M., Yoshida M., Ohnishi-Kameyama M., Shinmoto H. (2007). Ergosterol peroxide from an edible mushroom suppresses inflammatory responses in RAW264.7 macrophages and growth of HT29 colon adenocarcinoma cells. Br. J. Pharmacol..

[35] Ganeshpurkar A., Pardhi P., Bhadoriya S.S., Jain N., Rai G., Jain A.P. (2015). Antioxidant Potential of White Oyster Culinary-Medicinal Mushroom, *Pleurotus florida* (Higher Basidiomycetes). Int. J. Med. Mushrooms.

[36] Shao H.J., Jeong J.B., Kim K.J., Lee S.H. (2015). Anti-inflammatory activity of mushroom-derived hispidin through blocking of NF-κB activation. J. Sci. Food Agric..

[37] O’Callaghan Y.C., O’Brien N.M., Kenny O., Harrington T., Brunton N., Smyth T.J. (2015). Anti-inflammatory effects of wild Irish mushroom extracts in RAW264.7 mouse macrophage cells. J. Med. Food.

[38] Phan C.W., David P., Naidu M., Wong K.H., Sabaratnam V. (2015). Therapeutic potential of culinary-medicinal mushrooms for the management of neurodegenerative diseases: Diversity, metabolite, and mechanism. Crit. Rev. Biotechnol..

[39] Beelman R.B., Kalaras M.D., Richie J.P. (2019). Micronutrients and Bioactive Compounds in Mushrooms: A Recipe for Healthy Aging?. Nutr. Today.

[40] Kalaras M.D., Richie J.P., Calcagnotto A., Beelman R.B. (2017). Mushrooms: A rich source of the antioxidants ergothioneine and glutathione. Food Chem..

[41] Ey J., Schömig E., Taubert D. (2007). Dietary sources and antioxidant effects of ergothioneine. J. Agric. Food Chem..

[42] Jang J.H., Aruoma O.I., Jen L.S., Chung H.Y., Surh Y.J. (2004). Ergothioneine rescues PC12 cells from beta-amyloid-induced apoptotic death. Free Radic. Biol. Med..

[43] Halliwell B., Cheah I.K., Tang R.M.Y. (2018). Ergothioneine—A diet-derived antioxidant with therapeutic potential. FEBS Lett..

[44] Dolan H., Crain B., Troncoso J., Resnick S.M., Zonderman A.B., Obrien R.J. (2010). Atherosclerosis, dementia, and Alzheimer disease in the Baltimore Longitudinal Study of Aging cohort. Ann. Neurol..

[45] Yoo K.D., Park E.S., Lim Y., Kang S.I., Yoo S.H., Won H.H., Kim Y.H., Yoo I.D., Yoo H.S., Hong J.T. (2012). Clitocybin A, a novel isoindolinone, from the mushroom *Clitocybe aurantiaca*, inhibits cell proliferation through G1 phase arrest by regulating the PI3K/Akt cascade in vascular smooth muscle cells. J. Pharmacol. Sci..

[46] Iadecola C. (2014). Hypertension and dementia. Hypertension.

[47] Ganeshpurkar A., Kohli S., Rai G. (2014). Antidiabetic potential of polysaccharides from the white oyster culinary-medicinal mushroom *Pleurotus florida* (higher basidiomycetes). Int. J. Med. Mushrooms.

[48] Yoon K.N., Alam N., Shim M.J., Lee T.S. (2012). Hypolipidemic and antiatherogenesis effect of culinary-medicinal pink oyster mushroom, *Pleurotus salmoneostramineus* L. Vass. (higher Basidiomycetes), in hypercholesterolemic rats. Int. J. Med. Mushrooms.

[49] Khursheed R., Singh S.K., Wadhwa S., Gulati M., Awasthi A. (2020). Therapeutic potential of mushrooms in diabetes mellitus: Role of polysaccharides. Int. J. Biol. Macromol.

[50] Tian Y., Nichols R.G., Roy P., Gui W., Smith P.B., Zhang J., Lin Y., Weaver V., Cai J., Patterson A.D. (2018). Prebiotic effects of white button mushroom (*Agaricus bisporus*) feeding on succinate and intestinal gluconeogenesis in C57BL/6 mice. J. Funct. Foods.

[51] Alghamdi A. (2023). Structural and Functional Brain Changes Associated with Vitamin B12 Deficiency using Magnetic Resonance Imaging: A Systematic Review and Meta-analysis. Curr. Med. Imaging.

[52] Watanabe F., Yabuta Y., Bito T., Teng F. (2014). Vitamin B12-Containing Plant Food Sources for Vegetarians. Nutrients.

